# Environmental Risk Factors for Childhood Inflammatory Bowel Diseases: A Multicenter Case-Control Study

**DOI:** 10.3390/children9030438

**Published:** 2022-03-21

**Authors:** Mohammed Hasosah, Wafaa Alhashmi, Renad Abualsaud, Anas Alamoudi, Afnan Aljawad, Mariam Tunkar, Nooran Felemban, Ahmed Basalim, Muhammad Khan, Aziz Alanazi, Ali Almehaidib

**Affiliations:** 1 Pediatric Gastroenterology Department, King Saud bin Abdulaziz University for Health Sciences, King Abdullah International Medical Research Center (KAIMRC), National Guard Hospital, Jeddah 11481, Saudi Arabia; azizalanazi3@gmail.com; 2 College of Medicine, King Abdulaziz University, Jeddah 11481, Saudi Arabia; wafaahani.alhashmi@gmail.com (W.A.); rabualsaud@hotmail.com (R.A.); 3 College of Medicine, King Saud bin Abdulaziz University for Health Sciences, Jeddah 11481, Saudi Arabia; anasalamoudi99@gmail.com (A.A.); tunkar010@ksau-hs.edu.sa (M.T.); noranfel@gmail.com (N.F.); 7mato1419.ab@gmail.com (A.B.); annu250@hotmail.com (M.K.); 4 Department of Pediatric Gastroenterology, King Faisal Specialist Hospital & Research Center, Riyad 11211, Saudi Arabia; afaljawad@kfshrc.edu.sa (A.A.); mehaidib@kfshrc.edu.sa (A.A.)

**Keywords:** Crohn’s disease, inflammatory bowel diseases, environmental risk factors, ulcerative colitis

## Abstract

Objective: Multiple environmental factors can be linked to the development of inflammatory bowel disease (IBD). With an increase in the cases of IBD, the objective of this research is to investigate environmental risk factors for IBD in the Saudi population. Methods: A retrospective multicenter case–control study was performed among IBD children from 2009 to 2021.The variables analyzed to be the possible risk factors included their socioeconomic status, living and demographic characteristics, and lifestyle related to IBD. The questionnaire included a list of IBD risk factors that was given to the control and the patient group. For every variable, the 95% confidence interval (CI) and odds rations were also estimated. Results: There were 335 individuals considered in this study: 168 controls (50.1%) and 167 IBD patients (49.9%). Of these, 93 IBD patients (56%) had CD and 74 patients (44%) had UC. Most of participants were female (72.1%) and were aged above 10 years (51.5%). Vaginal delivery (OR 0.551, 95% CI: 1.59–4.14), age above 10 years (OR 1.040, 95% CI: 1.012–1.069), deficient fruit intake (OR 2.572, 95% CI: 1.59–4.14), no exposure to antibiotics (OR 2.396, 95% CI: 1.51–3.81), appendectomy (OR 2.098, 95% CI: 1.87–2.35), less physical activity (OR 2.033, 95% CI: 1.05–3.93) and gastroenteritis admissions > 2 times/year (OR 0.107, 95% CI: 0.037–0.311) were the risk factors for IBD. These factors depicted a more significant link with CD than UC (*p* < 0.05). Interestingly, sleep disturbance was estimated to be a CD risk factor (adjusted OR: 3.291, 95% CI = 0.97–11.22). Pets in house was risk factor for UC (*p* < 0.001). Conclusions: This study highlights association between vaginal delivery, age above 10 years, deficient fruit intake, low physical activity, exposure to antibiotics, appendectomy, and frequent gastroenteritis admissions as risk factors for IBD. Knowledge of these risk factors can help pediatricians to prospectively identify patients at risk of environmental exposure.

## 1. Introduction

Inflammatory bowel diseases (IBD), including ulcerative colitis (UC) and Crohn’s disease (CD) are chronic idiopathic illnesses of gastrointestinal tracts. Although it is still not known how IBD progresses, an underlying mechanism is reported to be a de-regulated host immune response to intestinal flora, and is genetically susceptible in humans [1].

Food additives, an improper diet, water pollution, air pollution, stress, infection, and related environmental factors were also investigated in the studies related to IBD and other autoimmune diseases. Collectively, all of the factors are called the exosomes [2]. Exposome are used to evaluate the exposure of environmental factors from birth to death, where some exposure to the Western lifestyle was considered to be chronic and cause metabolic inflammation [3]. So far, the strongest risk factor has been identified as having a family history of the disease. Familial correlation can be explained with either genetic factors or shared putative environmental risk factors [4]. However, an improper diet, exposure to antibiotics, the use of inflammatory non-steroidal medicines, contraceptives, and smoking were mainly suggested as environmental factors of IBD in the Western world [5]. In Brazil, a multicenter case–control study demonstrated that environmental risk factors, such as low income, lack of immunization, history of bowel infection, and family history of IBD, were linked to IBD development [6]. Another Italian case–control study determined that using antibiotics often throughout one’s infant years was linked with an increased chance of contracting IBD later in life [7]. A systematic analysis conducted via a meta-analysis of 35 studies deduced that breastfeeding influenced the development of ulcerative colitis and Crohn’s disease. Infants who were breastfed have a lower risk of developing both diseases [8].

A case–control study was carried out on an adult Saudi population to find the relationship between IBD development and the exposure to environmental risk factors. The results reported that there were associations between IBD and vaccinations in early life, educational background, and oral contraceptives for maternal use [9]. In Saudi Arabia, cases of IBD among children showed a trend of increased annual mean incidence, but the risk factors of IBD were not reported in children [10].

To the best of our knowledge, no case–control multicenter studies were conducted in children regarding pediatric IBD and environmental exposures. To enhance our knowledge of exposomes and the actions taken for the factors, in addition to the rising trend of IBD in the Saudi population, we aim to examine the link between the risk of childhood IBD and environmental exposure.

## 2. Methods

### 2.1. Study Population

A hospital-based retrospective case–control study was employed. We performed this study to examine an association between the risk of childhood IBD and environmental exposures in three tertiary hospitals in Jeddah and Riyadh, Saudi Arabia, during the period 2009–2021. Medical records of the selected centers were reviewed. The included children were aged from 0 to 16 years with diagnosed IBD. A combination of radiologic, histopathologic, endoscopic, and clinical assessments was conducted to diagnose IBD. Additional classification of UC and CD was conducted based on the “European Society of Pediatric Gastroenterology, Hepatology and Nutrition” (ESPGHAN) Working Group report (also called “the Porto criteria”). People suffering from irritable bowel syndrome or self-reported IBD or immunodeficiency were not included in this study.

### 2.2. Data Collection

**Figure 1:**
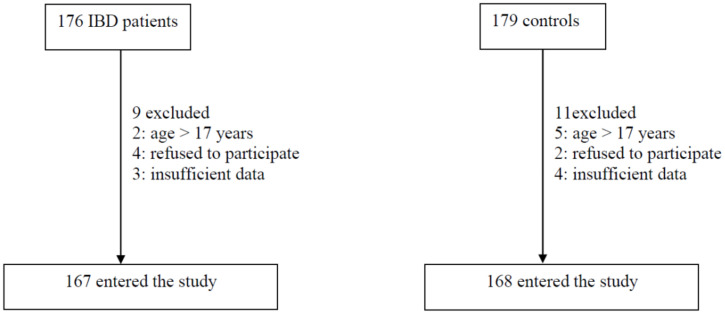
Flowchart reporting the process of patient selection.

Our sample size was 335 participants. Our targeted population for cases was patients from the IBD cohort of National Guard Hospitals (Jeddah and Riyad) and King Faisal Hospital (Riyadh). Controls were selected through convenience sampling technique from patients visiting outpatient clinics. Flowchart reporting of the process of patient selection is shown in Figure 1. Children with IBD (cases) and their matched controls were identified from our database. The user-friendly questionnaire was created to evaluate the demographic, socioeconomic characteristics and exposure variables to IBD. A group of pediatric gastroenterologists piloted the survey, initially built by the 2 pediatric gastroenterologists. Then, according to the reproducibility, validity, as well as question value, the survey was revised. A total of 10 pediatric gastroenterologists and five pediatric practitioners reviewed the original pool of items for content and ease of understanding; based on the findings of the pilot, modifications and adjustments were performed (Cronbach’s alpha = 0.8). The questionnaire was distributed through direct communication (face-to-face) as well as the online website, SurveyMonkey, over a three-month period from September 2021 to December 2021.

Variables that were obtained from each patient included: gender, age, BMI, diagnosis, fast foods, breastfeeding, exposure to antibiotics, parent smoking, exposure to infection, vaccination status, housing, family incomes, water pollution (assessment of water pollution was measured by the turbidity of water: temperature of water, the electrical conductivity or conductivity of water bodies, dissolved mineral content indicative of water quality for drinking), appendectomy, tonsillectomy, stress, anxiety, sleep disturbances, physical activity performance, pets in house, type of delivery, respiratory infection admission > 2 times/year, gastroenteritis admission > 2 times/year, bed sharing, diseases during pregnancy, drinking tap water, consanguinity, cow milk allergy, daily fruit intake (fruit intake includes any type of fruits in any amount; this measure was compared to zero fruit intakes), vitamin D intake, mother’s educational level, father’s education level. Level of education was divided into two levels: high (obtained a high school (secondary school) diploma or equivalent certificate) and low (obtained no school diploma). Each variable was assessed in both cases and controls. Information on presence or absence of family history of IBD (particularly parent with IBD or sibling with IBD) was not collected.

### 2.3. Statistical Analysis

Associations of health status, socio-demographic factors, and potential exposure variables of IBD (lifestyle and environmental conditions) among children were examined with univariate analysis. Backward step-wise method was followed to conduct the multivariate analysis; in this study, the final model was comprised of only those variables that were found to be statistically significant in the univariate analysis. The association was expressed with 95% of confidence intervals (CI) and odds ratios (OR). SPSS software (for Windows V.13) was used for data analysis. The demographic data were compared using the Wilcoxon rank-sum test or the Fisher’s exact test wherever suitable. For IBD, the risk factors were examined via Cox regression analysis. The 2-tailed tests show a statistical significance of *p* < 0.05. An association between IBD (univariate analysis) and epidemiologic factors was estimated. The research was approved by “Ethics and Research Committee of the National Guard Health Affairs, King Abdullah International Medical Research Center” (NRJ21J/199/08). Well-informed and written consent forms were completed by the parents or the guardians of the children taking part in the survey. The data were kept confidential. The data were in a secured data base electronic system with limited access and could only be accessed by the authors. No names or ID numbers were required to complete the data collection form.

## 3. Results

### 3.1. Characteristics of the Study Population

The study population included 335 individuals: 167 IBD patients (49.9%) and 168 controls (50.1%). Of these, 93 IBD patients (56%) had CD and 74 patients (44%) had UC. Most of participants were female (72.1%) and their age >10 years (51.5%). Other demographics of the study participants are shown in Table 1. Among the demographic characteristics, only age group > 10 years old (OR 1.040; 95% CI 1.012–1.069, *p* < 0.0001) showed a significant relationship with IBD and control patients. The characteristics of the cases and controls are described in Table 2.

### 3.2. Risk Factors for IBD

A total of six risk factors (listed in Table 2) for IBD were identified with the multivariate logistic regression analysis: newborn vaginal delivery (OD = 0.551, 95% CI = 0.348–0.874), deficient fruit intake (OD = 2.572, 95% CI = 1.59–4.14), exposure to antibiotics (OR = 2.396, 95% CI = 1.51–3.81), appendectomy (OD = 2.098, 95% CI = 1.87–2.35), less physical activity (OR = 2.033, 95% CI = 1.05–3.93) and gastroenteritis admissions > 2 times/year (OR = 0.107, 95% CI = 0.037–0.311) were the risk factors for IBD. Deficient fruit intake had a 2.5-fold higher likelihood of IBD compared with healthy fruit intake (Table 2). The other variables, which included obesity, consumption of fast foods, breastfeeding, vaccination status, parent smoking, type of housing, bed sharing, water pollution, tonsillectomy, 1st degree consanguinity, and parent education level, were not found to be significant for IBD progression factors.

Interestingly, sleep disturbance was estimated as the CD risk factor (adjusted OR: 3.291, 95% CI = 0.97–11.22). Although vitamin D intake failed to reach statistical significance with regard to the association with an increased risk of IBD in the univariate analysis (unadjusted OR = 0.707; 95% CI, 0.46–1.09), this association was statistically significant in a multivariate model adjusting for IBD types (adjusted OR = 2.574; 95% CI, 1.13–5.85). The risk factors included vaginal delivery, deficient fruit intake, less physical activity, exposure to antibiotics, appendectomy, family stress, and gastroenteritis admissions > 2 times/year showed a more significant link to CD than UC (*p* < 0.05). Having pets in the house was also deduced to have a more significant link to UC than CD (*p* < 0.001). Other risk factors are shown in Table 3. The age of diagnosis of IBD, gastroenteritis admissions > times/year, pets in house, type of delivery and other risk factors among the type of IBD are shown in Table 4.

## 4. Discussion

To the best of our knowledge, this is the first pediatric study in Saudi Arabia examining the link between childhood IBD and environmental exposures. The exact cause of IBD is obscure, but it is almost certainly caused by complex interactions between environmental exposures and genetics [11]. In Saudi Arabia, the incidence of pediatric IBD shows a trend of increased annual mean incidence, but risk factors were not reported [10]. Our study findings showed that the age group of IBD is mainly more than ten years. These findings are consistent with a study conducted by Buderus et al. [12], where more than three-quarters of IBD cases were reported among children aged above ten years, whereas 23.2% who had the disease were younger than ten years. 

The meta-analysis and a systematic review reported that no such significant difference exists between the IBD risk in children born vaginally as compared with a cesarean birth, and vaginal delivery was not an IBD risk factor [13]. The present paper reported that vaginal delivery was a significant IBD risk factor. The study by Dominguez-Bello et al. [14], combined with our observation, suggests that infants born via vaginal delivery acquire bacterial flora that are the same as their mother’s vaginal bacterial communities, developing an early colonization of Lactobacilli in these infants. The explanation for this finding is that delivery mode has an effect on the colonization of commensal flora in the gut, and vaginal delivery plays a role as risk factor in IBD.

Consistent with previous studies, appendectomy was significantly discovered to be an IBD risk factor in our study, involving CD rather than UC [15,16,17]. 

Our data show that tonsillectomy has no association with the risk of IBD progression. This is inconsistent with a meta-analysis and systematic review, which found that tonsillectomy is associated with a higher risk of developing CD.

There is also no proof that suggests that tonsillectomy exerts a protective impact on UC progression [18]. It is unclear why our patients with tonsillectomy were not linked to an increased IBD risk, possibly because of the interplay of gastrointestinal bacteria, genetic predisposition, and gut immunity. Another explanation is that the data collected were only limited to three centers.

Therefore, the conclusion of this study is to be interpreted with caution due to the sample size considered, which may result in an underestimation of the strength of the association between IBD, as well as its risk factors. Dietary fat was found to play a part in IBD pathogenesis, in contrast to previous studies that individuals who consumed a diet of fast food and high sugar with less fiber exhibited a high risk of developing IBD [19,20]. The habit of consuming fast food in our patients did not reach statistical significance as a risk factor for IBD. One possible explanation is that lifestyle-related factors were also self-reported by the patients considered in the study.

In our findings, children with a high fruit intake had a significantly lower risk for IBD than those with more deficient fruit intake. Studies showed that a higher consumption of dietary fibers, especially soluble fibers in the form of fruits and vegetables, decreased the incidence of IBDs [21,22]. One possible explanation for this is that the intestinal microbiota convert soluble fibers into short-chain fatty acids, which could inhibit the transcription of pro-inflammatory mediators in the body [23], in contrast to Kugathasan et al. [24] and Long et al. [25], who reported that IBD incidence in obese children is higher than in children with an average body mass index. The present study reported that a higher prevalence of IBD was in obese children, but it was statistically insignificant as a risk factor in IBD.

Another important risk factor for IBD is an infection. Many studies demonstrated an increased incidence of IBD with individuals exposed to microorganisms [26,27]. The findings of García Rodríguez et al. [28] were considered with observation in the patients of our study and suggest that frequent episodes of acute gastroenteritis could play a role in the exacerbation and/or development of IBD. Our study found that IBD incidence was higher among children with gastroenteritis admission more than two times a year.

It is hypothesized that stress activates neural pathways in the hypothalamus that lead to the sympathetic and parasympathetic nervous systems, thus causing an impairment in gastrointestinal motility [29]. Our results support the findings that family stress and anxiety were found to be the main IBD risk factors for CD. This is similar to the study by Giannakopoulos et al. [30] that stressful child anxiety, life events, and mental health symptoms of the parents could trigger the development of IBD in children. The study by Jakobsen et al., combined with the observation in our study, suggests that stressful events and gastrointestinal infection during childhood were identified as risk factors for developing pediatric IBD [31].

The overuse of antibiotics was associated with IBD development, which was linked with the interference of the normal gut enteric bacteria [32]. Our logistic regression analysis model showed no relationship between the overuse of antibiotics and IBD.

The present paper reported that less physical activity was found to be a risk factor before the development of IBD. This result is similar to the finding that exercise and physical activity boosts immune response and reduces pro-inflammatory cytokines [33]. However, IBD, specifically the presence of its active symptoms, impairs the ability of patients to participate in sports or exercise [34]. Ananthakrishnan reported that disturbance in sleep is linked to a higher risk of disease progression in CD [35]. Our model of a logistic regression analysis demonstrated that disturbance in sleep is considered as a potential factor of risk for IBD progression.

Smoking is an independent predictive factor for IBD progression among adults, but it is not clear how passive smoking plays a part in pathophysiology of IBD [36]. It may be argued that the inhalation of smoke in children may cause immunosuppression, thus contributing to IBD progression [37]. In our study, the passive smoking variable was not significantly found to be an IBD-related risk factor. For IBD in our patients, breastfeeding was not concluded to be a risk factor. However, considering the relatively small number of studied cases, this association must be cautiously interpreted and verified by trials before any causal importance can be adhered to it.

The strength of this study was that it was the first pediatric multicenter conducted in Saudi Arabia that examined the link between childhood IBD and environmental exposures. However, our research is limited in several aspects. The sample is only considered from three centers of Saudi Arabia; hence, the results of the study must be cautiously interpreted as the limited sample size might result in an underestimation of any associations that exist between risk factors and IBD. Second, it was a retrospective study with the small number of cases in some categories. In addition, the age group above 10 years was predominant, which might contribute to the overinflation of the proportion of age group. Third, some data retrieved from parents could be subjected to recall bias. Fourth, the data collected were limited for estimating the variables during this study and not during diagnosis of IBD, thus limiting the potential effect of some variables. In addition, there were no validated measures to assess the variables. Finally, genetic predisposition is well-documented as a major risk factor for the development of IBD, and the data of genetic predisposition in our study are not available.

In summary, the occurrence of IBD is increasing in the Saudi pediatric population. Thus, understanding and recognizing these risk factors in Saudi children is crucial for characterizing environmental factors and designing targeted and cost-effective strategies to prevent this disease.

## 5. Conclusions

This study highlights the association between vaginal delivery, deficient fruit intake, low physical activity, exposure to antibiotics, appendectomy, and frequent gastroenteritis admissions as risk factors for IBD. The knowledge of these risk factors can help pediatricians prospectively identify patients at risk from environmental exposures. Therefore, future longitudinal prospective studies are required to confirm the association of risk factors in this field of research.

## Figures and Tables

**Table 1:** Demographic and characteristics of the study participants.

	*n* = 335	%
Diagnosis		
Control	168	50.1
IBD	167	49.9
Age		
<3 years	21	6.3
3–6 years	34	10.1
7–10 years	92	27.5
>10 years	188	56.1
Gender (*n* = 334)		
Male	172	51.5
Female	162	48.5
BMI (*n* = 334)		
Obesity	44	13.2
No obesity	290	86.8
Housing (*n* = 334)		
Rural	41	12.3
Urban	293	87.7
Family incomes (*n* = 333)		
<5000 SR	53	15.9
5000 SR	40	12.0
>5000 SR	240	72.1
Mother education level		
High	191	57.0
Low	144	43.0
Father education level		
High	198	59.1
Low	137	40.9
Consanguinity 1st degree		
Yes	133	39.7
No	202	60.3
Breastfeeding (*n* = 333)		
1 month	66	19.8
<6 months	95	28.5
6–12 months	17	5.1
>12 months	155	46.5
Type of delivery (*n* = 333)		
Vaginal	220	66.1
Caesarean	113	33.9
Vaccination		
None	3	0.9
Completed	302	90.1
Partially completed	30	9.0
Parent Smoking (mother)		
Yes	7	2.1
No	328	97.9
Parent Smoking (father)		
Yes	111	33.1
No	224	66.9

BMI: body mass index, SR: Saudi Riyal.

**Table 2:** Logistic regression analysis of demographic characteristics and their association/non-association with IBD patients.

	Diagnosis							
	Control		IBD		OR	95% CI		*p*
	*n*	%	*n*	%				
Age (*n* = 335)					0.023	0.02	0.174	<0.001 *
<3 years	20	95.2%	1	4.8%				
3–6 years	32	94.1%	2	5.9%				
7–10 years	57	62.0%	35	38.0%				
>10 years	59	31.4%	129	68.6%				
Gender (*n* = 334)					0.714	0.464	1.099	0.125 *
Male	79	45.9%	93	54.1%				
Female	88	54.3%	74	45.7%				
BMI (*n* = 334)					0.655	0.344	1.247	0.196 *
Obesity	18	40.9%	26	59.1%				
No obesity	149	51.4%	141	48.6%				
Housing (*n* = 334)					0.835	0.433	1.607	0.588 *
Rural	19	46.3%	22	53.7%				
Urban	149	50.9%	144	49.1%				
Family incomes (*n* = 333)					1.238	0.682	2.922	0.440 *
<5000 SR	25	47.2%	28	52.8%				
5000 SR	17	42.5%	23	57.5%				
>5000 SR	126	52.5%	114	47.5%				
Mother education level					1.290	0.836	1.990	0.250
High	101	52.9%	90	47.1%				
Low	67	46.5%	77	53.5%				
Father education level					0.732	0.473	1.134	0.162
High	93	47.0%	105	53.0%				
Low	75	54.7%	62	45.3%				
Consanguinity 1st degree					0.732	0.473	1.134	0.162
Yes	67	50.4%	66	49.6%				
No	101	50.0%	101	50.0%				
Breastfeeding (*n* = 333)					0.83	0.466	1.479	0.340 *
1 month	35	53.0%	31	47.0%				
<6 months	46	48.4%	49	51.6%				
6 months	12	70.6%	5	29.4%				
>6 months	75	48.4%	80	51.6%				
Type of delivery (*n* = 333)					0.551	0.348	0.874	0.011
Vaginal	100	45.5%	120	54.5%				
Caesarean	68	60.2%	45	39.8%				
Vaccination					cannot be calculated			0.562 **
None	0	0.0%	3	100.0%				
Completely	150	49.7%	152	50.3%				
Partially complete	18	60.0%	12	40.0%				
Parent Smoking (mother)					0.741	0.163	3.362	0.723 **
Yes	3	42.9%	4	57.1%				
No	165	50.3%	163	49.7%				
Parent Smoking (father)					0.914	0.580	1.441	0.699 *
Yes	54	48.6%	57	51.4%				
No	114	50.9%	110	49.1%				

OR: odds ratio, CI: Confidence interval. * Chi-squared test ** Fisher’s exact test.

**Table 3:** Logistic regression analysis of risk factors and their association/non-association with IBD patients.

	Diagnosis							
	Control		IBD		OR	95% CI		*p*
	*n*	%	*n*	%				
Fast food					1.181	0.741	4.40	0.422 *
Daily	16	38.1%	26	61.9%				
Weekly	97	51.6%	91	48.4%				
Monthly	35	52.2%	32	47.8%				
Never	20	52.6%	18	47.4%				
Fruit intake					2.572	1.597	4.142	<0.001 *
Yes	71	65.7%	37	34.3%				
No	97	42.7%	130	57.3%				
Food allergy					0.733	0.392	1.372	0.330 *
Yes	20	43.5%	26	56.5%				
No	148	51.2%	141	48.8%				
Cow milk allergy					0.545	0.179	1.663	0.280 *
Yes	5	35.7%	9	64.3%				
No	161	50.5%	158	49.5%				
Exposure to antibiotics					2.396	1.507	3.808	<0.001 *
None	61	38.4%	98	61.6%				
Monthly	22	64.7%	12	35.3%				
Yearly	85	59.9%	57	40.1%				
Water Pollution					1.200	0.359	4.011	0.767
Yes	6	54.5%	5	45.5%				
No	162	50.0%	162	50.0%				
Appendectomy					2.098	1.871	2.353	<0.001
Yes	0	0.0%	14	100.0%				
No	168	52.3%	153	47.7%				
Tonsillectomy					0.653	0.312	1.370	0.257
Yes	13	40.6%	19	59.4%				
No	155	51.2%	148	48.8%				
Family Stress, anxiety					0.259	0.155	0.433	<0.001
Yes	27	27.6%	71	72.4%				
No	141	59.5%	96	40.5%				
Sleep disturbance					0.242	0.125	0.470	<0.001
Yes	13	23.2%	43	76.8%				
No	155	55.6%	124	44.4%				
Physical activity					2.033	1.053	3.927	<0.001
None	36	32.1%	76	67.9%				
1–2 times/week	19	47.5%	21	52.5%				
3 times/week	5	25.0%	15	75.0%				
>3 times/week	108	66.3%	55	33.7%				
Pets in house					0.370	0.193	0.707	0.002
Yes	15	30.0%	35	70.0%				
No	153	53.7%	132	46.3%				
Vit D intake					0.707	0.460	1.086	0.113
Yes	77	45.8%	91	54.2%				
No	91	54.5%	76	45.5%				
Respiratory infection admissions > 2 times/year					0.820	0.406	1.655	0.579
Yes	16	45.7%	19	54.3%				
No	152	50.7%	148	49.3%				
Gastroenteritis admissions > 2 times/year					0.107	0.037	0.311	<0.001
Yes	4	11.4%	31	88.6%				
No	164	54.7%	136	45.3%				
Bed share					0.877	0.500	1.540	0.649
Yes	28	47.5%	31	52.5%				
No	140	50.7%	136	49.3%				
Diseases during pregnancy					1.202	0.698	2.069	0.506
Yes	35	53.8%	30	46.2%				
No	132	49.3%	136	50.7%				

* Chi-squared test.

**Table 4:** Difference between CD and UC according to risk factors.

	Diagnosis						
	CD		UC		Normal		*p*
	*n*	%	*n*	%	*n*	%	
Age							<0.001 *
<3 years	1	4.8%	0	0.0%	20	95.2%	
3–6 years	0	0.0%	2	5.9%	32	94.1%	
10 years	19	20.7%	16	17.4%	57	62.0%	
>10 years	73	38.8%	56	29.8%	59	31.4%	
Fruit intake							<0.001 *
Yes	19	17.6%	18	16.7%	71	65.7%	
No	74	32.6%	56	24.7%	97	42.7%	
Exposure to antibiotics							0.001 *
None	56	35.2%	42	26.4%	61	38.4%	
Monthly	8	23.5%	4	11.8%	22	64.7%	
Yearly	29	20.4%	28	19.7%	85	59.9%	
Appendectomy							<0.001 **
Yes	10	71.4%	4	28.6%	0	0.0%	
No	83	25.9%	70	21.8%	168	52.3%	
Family stress, anxiety							<0.001 *
Yes	39	39.8%	32	32.7%	27	27.6%	
No	54	22.8%	42	17.7%	141	59.5%	
Sleep disturbance							<0.001 *
Yes	25	44.6%	18	32.1%	13	23.2%	
No	68	24.4%	56	20.1%	155	55.6%	
Physical activity							<0.001 *
None	38	33.9%	38	33.9%	36	32.1%	
1–2 times/week	11	27.5%	10	25.0%	19	47.5%	
3 times/week	8	40.0%	7	35.0%	5	25.0%	
>3 times/week	36	22.1%	19	11.7%	108	66.3%	
Pets in house							0.001 *
Yes	15	30.0%	20	40.0%	15	30.0%	
No	78	27.4%	54	18.9%	153	53.7%	
Type of delivery							0.036 *
Vaginal	65	29.5%	55	25.0%	100	45.5%	
Caesarean	26	23.0%	19	16.8%	68	60.2%	
Gastroenteritis admissions > times/year							<0.001 *
Yes	16	45.7%	15	42.9%	4	11.4%	
No	77	25.7%	59	19.7%	164	54.7%	

* Chi-squared test.

## Data Availability

This study did not report any data.
